# Supplementary material for: Four Genetic Polymorphisms of Lymphotoxin-Alpha Gene and Cancer Risk: A Systematic Review and Meta-Analysis
Source: PLoS One. 2013 Dec 12;8(12):e82519. doi: 10.1371/journal.pone.0082519 (PMC3861395; doi:10.1371/journal.pone.0082519)
Supplement: Checklist S1 — PRISMA checklist. (DOC) [file pone.0082519.s001.doc]

| **Section/topic** | **#** | **Checklist item** | **Reported on page #** |
| --- | --- | --- | --- |
| **TITLE** | | | Title |
| Title | 1 | Four genetic polymorphisms of lymphotoxin-alpha gene and cancer risk: a systematic review and meta-analysis | Title |
| **ABSTRACT** | | | Abstract |
| Structured summary | 2 | Lymphotoxin-alpha (LTA) is a pro-inflammatory cytokine that plays an important role in the inflammatory and immunologic response. Numerous studies have shown *LTA* polymorphisms as risk factors for cancers, but the results remain inconclusive. The goal of the present meta-analyses is to establish the associations between cancers and four *LTA* variants (rs1041981, rs2239704, rs2229094 and rs746868). A total of 30 case-control studies involving 58,649 participants were included in the current meta-analyses. Our results showed significant associations with increased cancer risk for rs1041981 (odd ratio (OR) = 1.15, 99% confidential interval (CI) = 1.07-1.25, P < 0.0001, I^2^ = 12.2%), rs2239704 (OR = 1.08, 99% CI = 1.01-1.16, P = 0.021, I^2^ = 0.0%) and rs2229094 (OR = 1.28, 99% CI = 1.09-1.50, P = 0.003, I^2^ = 0.0%). No evidence was found for the association between rs746868 and cancer risk (OR = 1.01, 99% CI = 0.93-1.10, P = 0.771, I^2^ = 0.0%). Subgroup meta-analysis suggested that rs2239704 was likely to increase the risk of hematological malignancy (OR = 1.10, 99% CI = 1.01–1.20, P = 0.023, I^2^ = 0.0%), and rs2229094 was specific for the increased risk of adenocarcinoma (OR = 1.33, 99% CI = 1.11-1.59, P = 0.002, I^2^ = 0.0%). In conclusion, our meta-analyses suggested that the *LTA* rs1041981, rs2239704 and rs2229094 polymorphisms contributed to the increased risk of cancers. Future functional studies were needed to clarify the mechanistic roles of the three variants in the cancer risk. | Abstract |
| **INTRODUCTION** | | | Introduction |
| Rationale | 3 | With the high prevalence and mortality rate, cancers have become one of the main causes of morbidity and mortality worldwide . There are over 200 different known cancers in humans, and the mechanisms of cancer pathogenesis remain obscure . Large epidemiologic and clinical studies have illustrated that inflammation may be associated with the development of cancers . Increasing evidence suggests that predisposition to cancer is associated with cytokines , such as tumor necrosis factors (TNF) .  Lymphotoxin-alpha (LTA) is a pro-inflammatory cytokine belong to the TNF family which plays an important role in the inflammatory and immunologic response . LTA is a product of stimulated T cells , and it can help communicate lymphocytes and stromal cells and subsequently eliciting cytotoxic effects on cancer cells . *LTA* gene is located on the 6p21.3 that harbors the class III region of the major histocompatibility complex (MHC) locus . Genetic variations of inflammation-related genes are shown to alter both the regulation of the inflammatory response and modulation of susceptibility to radiation-induced normal tissue damage . *LTA* gene polymorphisms are shown to be associated with the inflammatory and immunomodulatory diseases including cancers . The association is significant between *LTA* polymorphisms and cancers including gastric and breast cancers in Asians, colorectal cancer in Germans, and Non-Hodgkin Lymphoma (NHL) in Europeans . However, discrepancies remain for the association of *LTA* polymorphisms with cancers in different ethnic groups .  Four *LTA* polymorphisms (rs1041981:Thr26Asn; rs2239704; rs2229094: Cys13Arg; rs746868) have been extensively investigated as potential risk factor for cancer. These mutations may exert possible regulatory regulation on the gene expression and the level of cytokine production . Single nucleic polymorphism (SNP) rs1041981 (Thr26Asn) is associated with the transcriptional regulation of LTA, which may activate the lymphocytes and induce apoptosis . SNP rs2239704 can modulate both LTA levels and subsequent inflammatory response to pathogens . SNP rs746868 is shown to be in high linkage disequilibrium (LD) with rs1041981 (r^2^ > 0.8) . SNPs rs746868 and rs2239704 are significant predictive variables of LTA protein production . Haplotype of SNP rs2229094 is shown to be associated with altered LTA expression and increased levels of vascular- and autoimmune-mediated inflammation . | Introduction |
| Objectives | 4 | The associations of *LTA* variations with cancer risk have been evaluated by several case-control studies . Previous studies have suggested that the functional polymorphism rs909253 is associated with gastric and breast cancers in Asians. Although the four SNPs (rs1041981, rs2239704, rs2229094 and rs746868) are present in high LD with rs909253 , inconsistent results of the four SNPs and cancer risk are observed for different cancers in Asian, North American and European populations . In the current study, we perform a comprehensive meta-analysis to evaluate the effects of the four functional SNPs (rs1041981, rs2239704, rs2229094 and rs746868) on cancer risk. | Introduction |
| **METHODS** | | | Materials and methods |
| Protocol and registration | 5 | no |  |
| Eligibility criteria | 6 | Articles enrolled in our meta-analyses met the following inclusion criteria: (1) evaluating the association between LTA polymorphism rs1041981, rs2239704, rs2229094 or rs746868 and cancer risk; (2) case-control study; (3) results with sufficient published data to estimate an OR with a 95% CI; (4) the genotype distribution in controls met Hardy-Weinberg equilibrium (HWE). | Inclusion criteria |
| Information sources | 7 | The literatures included in the analysis were obtained from the databases of PubMed, Web of Science, and China National Knowledge Infrastructure (CNKI). | Publication search |
| Search | 8 | The keywords applied the Medical Subject Headings (MeSH) in the US National Library of Medicine terms including “LTA”, “Lymphotoxin alpha”, “TNF-beta”, “polymorphism”, and “rs1041981”, “Thr26Asn”, “rs746868”, “rs2229094”, “Cys13Arg” or “rs2239704” paired with “cancer” or “tumor”. | Publication search |
| Study selection | 9 | Meanwhile, the manuscripts should be published in Chinese or English up to July 2013. | Publication search |
| Data collection process | 10 | Full texts were read to select the relevant information. The related articles in the MEDLINE as well as the reference lists of all retrieved studies were also checked for citations of other relevant publications that were not identified initially. | Publication search |
| Data items | 11 | Two investigators (YH and XY) independently extracted the data from all eligible publications basis on the selection criteria listed above. Any disagreements were established by discussion until a consensus was reached. If there were numerous publications from the same study group, the most complete and recent results were extracted. Useful information collected from each study comprise the first author’s surname, the year of publication, country, ethnic population, source of control groups (population- or hospital-based), cancer type, total sample size, genotyping method and the number of genotype distribution in cases and controls. | Data extraction |
| Risk of bias in individual studies | 12 | ORs and 99% CIs in the case-control studies were employed to assess the association between the *LTA* polymorphisms and cancer risk | Statistical analyses |
| Summary measures | 13 | The pooled ORs were performed under the additive, dominant, and recessive models. Z test was employed to estimate the significance of pooled OR. The departure of HWE for the genotype distribution in controls were analyzed by the Arlequin program (version 3.5) . The meta-analyses were performed using the Review Manger and Stata software (version 11.0, Stata Corporation, College Station, TX) . | Statistical analyses |
| Synthesis of results | 14 | A chi-square based Q-statistic test was calculated for the heterogeneity of studies in the meta-analysis . The inconsistency index (I^2^ statistic) was examined to judge the heterogeneity between studies . An I^2^ > 50% suggested a significant heterogeneity in the meta-analysis. Random-effect or fixed-effect models were used for, the meta-analysis with significant heterogeneity (I^2^ > 50%) or the one with minimal to moderate heterogeneity (I^2^ < 50%), respectively. | Statistical analyses |

Page 1 of 2

| **Section/topic** | **#** | **Checklist item** | **Reported on page #** |
| --- | --- | --- | --- |
| Risk of bias across studies | 15 | The Begg’s funnel plots and Egger regression test were used to evaluate the Publication bias . | Statistical analyses |
| Additional analyses | 16 | Subgroup meta-analyses were performed by cancer type, population, and source of control. A sensitivity analysis was also performed by excluding each study. The Begg’s funnel plots and Egger regression test were used to evaluate the Publication bias . A two-tailed P < 0.05 was considered statistically significant. | Statistical analyses |
| **RESULTS** | | | Results |
| Study selection | 17 | As shown in Figure 1, the literature search identified a total of 248 potentially relevant records. After reading the title or abstract, 50 studies concerning the association for the four *LTA* polymorphisms and cancer risk were considered for the following step. A total of 16 articles were excluded for insufficient genotyping information, or ineligible samples, or other *LTA* polymorphism, or duplicated studies. Finally, we collected 24 articles (including 30 study stages) focusing on the relationship of rs1041981, rs2239704, rs2229094 or rs746868 polymorphisms with cancer risk. | Literature flow |
| Study characteristics | 18 | Characteristics of enrolled studies were summarized in Table 1. Among the 24 eligible studies, 8 articles (including 11 stages with 7,483 cases and 11,938 controls) focused on the association between rs1041981 and cancers. A total of 10 studies , reported the association of cancers with rs2239704 including 13 stages among 6,049 cases and 7,621 controls. And 5 studies (including 9 stages) among 7,133 cases and 10,305 controls evaluated the contribution of rs2229094 to the risk of cancers. There were 7 articles (including 3,487 cases and 4,633 controls) involved with the association between rs746868 and cancers. All the records were collected from English publications. There were 7 Asian studies, 9 European studies, and 14 North American studies. Most of the cancer types were adenocarcinoma and hematological malignancy including gastric , breast , lung , prostate , and cervical cancers , colorectal adenoma , Non-Hodgkin Lymphoma (NHL) , Chronic Lymphocytic Leukaemia (CLL) and Testicular Germ Cell Tumors (TGCT) . The 30 selected studies in the meta-analysis included 13 studies with hospital-based controls, 17 studies with population-based controls. The controls were primarily population-based ones matched for ethnicity, age, gender or study region. | Study characteristics |
| Risk of bias within studies | 19 | As shown in Table 2, the genotype distribution of the four SNPs and the genotyping method of the collected studies were retrieved carefully. Genotype distributions of four polymorphisms in all the controls met HWE (P > 0.05). | Study characteristics |
| Results of individual studies | 20 | The results of individual studies are in Table 1. | Study characteristics |
| Synthesis of results | 21 | Meta-analysis of rs1041981  The main results of the meta-analysis for rs1041981 polymorphism were presented in Table 3. No heterogeneity existed in the involved studies (I^2^ = 16.0%, P = 0.29). The summary effect OR was 1.10 for G allele (99% CI = 1.04-1.16, P = 0.001, Figure 2). Subgroup analysis suggested that rs1041981 increased the risk of several types of cancer, such as adenocarcinoma (OR = 1.07, 99% CI = 1.01-1.14, P = 0.030, I^2^ = 0.08%), squamous carcinoma (OR = 1.19, 99% CI = 1.03-1.37, P = 0.018, I^2^ = 46.0%), hematological malignancy (OR = 1.73, 99% CI = 1.08-2.77, P = 0.023). The significant association were also found in multiple populations including Asians (OR = 1.18, 99% CI = 1.04-1.34, P = 0.010, I^2^ = 0.0%) and Europeans (OR = 1.07, 99% CI = 1.00-1.14, P = 0.047, I^2^ = 0.0%). In addition, the source analysis showed positive association in both hospital-based group (OR = 1.24, 99% CI = 1.09-1.42, P = 0.001, I^2^ = 0.0%) and population-based group (OR = 1.07, 99% CI = 1.00-1.14, P = 0.042, I^2^ = 0.0%, Table 3).  Meta-analysis result of rs2239704  Figure 3 showed the forest plot for the rs2239704 stratified by cancer type. Significantly increased cancer risk was found in the G versus A model based on the studies (OR = 1.08, 99% CI = 1.01-1.16, P = 0.021, I^2^ = 0.0%, Table 4). In the subgroup meta-analysis by cancer type, the rs2239704-G was observed with positive association with hematological malignancy (OR = 1.10, 99% CI = 1.01-1.20, P = 0.023, I^2^ = 0.0%, Table 4). Significant associations of rs2239704 with hematological malignancy cancers were also found in North American populations (OR = 1.09, 99% CI = 1.01-1.17, P = 0.025, I^2^ = 2.7%) and in the population-based group (OR = 1.08, 99% CI = 1.00-1.17, P = 0.044, I^2^ = 2.2%, Table 4).    Meta-analysis result of rs2229094  The main pooled data for rs2229094 polymorphism were listed in Table 5. For the overall data of the enrolled 9 study stages, significant association of rs2229094 polymorphism with cancer risk were shown on Figure 4 (OR = 1.28, 99% CI = 1.09-1.50, P = 0.003, I^2^ = 0.0%). In the additional analysis, significantly increased risks were observed in adenocarcinoma (OR = 1.33, 99% CI = 1.11-1.59, P = 0.002, I^2^ = 0.0%). In the stratified analysis by population, strong association of rs2229094 with adenocarcinoma cancers was found in Asians (OR = 1.92, 99% CI = 1.04-3.57, P = 0.038, I^2^ = 0.0%) and Europeans (OR = 1.26, 99%CI = 1.02-1.56, P = 0.029, I^2^ = 0.0%). Further subgroup analysis in recessive model, statistically associations were presented both hospital-based (OR = 1.92, 99% CI = 1.04-3.57, P = 0.038, I^2^ = 0.0%) and population based groups (OR = 1.24, 99% CI = 1.05-1.46, P = 0.010, I^2^ = 0.0%, Table 5).  Meta-analysis result of rs746868  There was no evidence of association between rs746868 and cancer risk (OR = 1.01, 99% CI = 0.93-1.10, P = 0.771, I^2^ = 0.0%, Supplement Table 1). Alternative genetic models and subgroup meta-analyses did not reveal any significant results (Table S 1). | Meta-analysis of rs1041981  Meta-analysis result of rs2239704  Meta-analysis result of rs2229094  Meta-analysis result of rs746868 |
| Risk of bias across studies | 22 | The Egger’s population bias plot indicated no visual publication bias in the meta-analysis (Figure 5, P = 0.084 for rs1041981; P = 0.602 for rs2239704; P = 0.433 for rs2229094; P = 0.343 for rs746868). | Sensitivity analyses and publication bias |
| Additional analysis | 23 | The sensitivity analyses were performed by excluding each study. The statistical significance of the results was not changed (data not shown). | Sensitivity analyses and publication bias |
| **DISCUSSION** | | | Discussion |
| Summary of evidence | 24 | In the systematic review and meta-analysis, we analyze 30 case-control studies among 58,649 participants to verify the association between the four SNPs and cancer risk. Our findings suggest that rs1041981, rs2239704 and rs2229094 are able to increase cancer risk by the significant association results under different genetic models. No evidence is found for the association between rs746868 and cancer risk.  In subgroup meta-analyses stratified by cancer type, our results show that rs2239704 and rs2229094 are likely to increase the risk of hematological malignancy and adenocarcinoma, respectively. Hematological malignancy is one cancer of uncontrolled proliferation of clonal B-cells, T-cell or NK-cell at different stages of maturations . Adenocarcinoma can arise in many tissues of the body including stomach, breast, pancreas, colon and so on . Both adenocarcinoma and hematological malignancy are multi-factorial diseases with complex interactions of genetic and environmental factors . Accumulating evidences have reported that genetic variations in immune genes are susceptibility loci for hematological malignancy . *LTA* rs2239704 polymorphism is the main predictor of LTA production in human B cells . Adenocarcinoma is the most common histologic type of cancer. The *LTA* variants are shown to be associated with cancer risks under different genetic models. These genetic models consist of additive (per variant allele vs. common allele), dominant (variant allele carriers vs. homozygotes for the common allele) and recessive (homozygotes for the variant allele vs. all others) genetic models . *LTA* rs1041981 is associated with a significantly lower presence of Japanese male lung cancer under the dominant model (CA+AA versus CC) ; *LTA* rs909253 is associated with high risk of Asian gastric cancer in the heterozygote comparison (GA versus AA) ; *LTA* rs2009658 is shown to be associated with significantly elevated risk of breast cancer among Caucasian women aged 45–64 years under the additive model (C versus G) . Our results showed that *LTA* rs1041981 and rs2239704 polymorphisms were correlated with cancer in three genetic models, suggesting an additive effect for these two polymorphisms on the risk of cancer. In contrast, rs2229094 was associated with the risk of adenocarcinoma in recessive model, implying a lack of contribution for the heterozygote to the risk of adenocarcinoma. The results could partly be attributed to different *LTA* polymorphisms that play different roles in different cancers and different populations. Additionally, the different tumors in humans may be generated by special carcinogenic mechanisms which would lead to multiple connections with one same genetic locus .  Our subgroup meta-analyses by ethnicity find that rs1041981 shows a negative relationship with cancers in Asians under the recessive model. SNP rs2239704 shows positive association in North Americans but not Europeans. Additionally, rs2229094 is associated with cancer in Asians but not in North Americans or Europeans. This might be explained by the different genotype and allele frequencies of these SNPs in subjects with different clinical characteristics, geographic distributions, and ethnic descent. Therefore, we could not exclude that the negative association in Europeans or North Americans may be due to a lack of power. Thus, matching criteria and selection bias and the stages of the cancers should be considered in the future case-control studies. Large sample size and various populations study would get more believable result in the future. | Discussion |
| Limitations | 25 | In the present study, we have collected a series of parameters (including cancer type, population, source of control, genotyping method, and matching condition) to yield reliable result in the meta-analyses. We require that the genotype distribution in the controls met HWE (P > 0.05). We also perform subgroup meta-analyses by the collected parameters to reduce the potential stratification among the involved case-control studies. However, there are some limitations in our meta-analysis should be mentioned. Firstly, our meta-analyses combine the genetic studies from various cancers that may introduce dramatic stratifications in the meta-analyses, although we have controlled several parameters. Secondly, Our results can’t tell the true causal variant of cancer risk from the three significant SNPs (rs1041981, rs2239704, rs2229094) since they are in high LD. Moreover, it is possible that the true causal variant may be other variant in high LD with them. Future investigation for the mechanistic roles of them is needed. Thirdly, there are only a few studies in the African populations. Future study in African populations needed to be performed in a large size cohort to investigate whether the negative results of subgroup meta-analysis in the African populations is due to a lack of power or genetic heterogeneity. | Discussion |
| Conclusions | 26 | In summary, the overall data of the present analyses suggest that three *LTA* variants (rs1041981, rs2239704 and rs2229094) can significantly increase the risk of cancers. Further well-designed studies in view of these variants are needed to explore their mechanistic roles in the pathogenesis of cancers. | Discussion |
| **FUNDING** | | | Funding |
| Funding | 27 | The research was supported by the grants from the National Natural Science Foundation of China (31100919), Natural Science Foundation of Zhejiang Province (LR13H020003), Medicine and health science and technology projects of Zhejiang province (2010KYB094, 2013KYB233), K. C. Wong Magna Fund in Ningbo University, Ningbo social development research projects (2011C50028, 2012C50027, 2012C50032), and Scientific Innovation Team Project of Ningbo (2011B82014), and Advanced key Scientific and Technological Programs of Ningbo (2011C51005). | Funding |

*From:*  Moher D, Liberati A, Tetzlaff J, Altman DG, The PRISMA Group (2009). Preferred Reporting Items for Systematic Reviews and Meta-Analyses: The PRISMA Statement. PLoS Med 6(6): e1000097. doi:10.1371/journal.pmed1000097

For more information, visit: **www.prisma-statement.org**.

Page 2 of 2
