# Supplementary material for: Four Genetic Polymorphisms of Lymphotoxin-Alpha Gene and Cancer Risk: A Systematic Review and Meta-Analysis
Source: PLoS One. 2013 Dec 12;8(12):e82519. doi: 10.1371/journal.pone.0082519 (PMC3861395; doi:10.1371/journal.pone.0082519)
Supplement: Table S1 — Meta-analysis of rs746868. (DOCX) [file pone.0082519.s002.docx]

Table S 1: Meta-analysis of rs746868

| Variables |  | Additive model (G versus A) | | Dominant model | | Recessive model | |
| --- | --- | --- | --- | --- | --- | --- | --- |
|  | N | P, OR(99% CI) | P_(Q-test)_, I^2^ | P, OR(99% CI) | P_(Q-test)_, I^2^ | P, OR(99% CI) | P_(Q-test)_, I^2^ |
| Total | 7 | 0.771, 1.01(0.93-1.10) | 0.99, 0.0% | 0.975, 1.00(0.89-1.12) | 0.99, 0.0% | 0.48, 1.05(0.92-1.20) | 0.99, 0.0% |
| **Source of control** |  |  |  |  |  |  |  |
| Hospital based | 4 | 0.659, 1.03(0.90-1.19) | 0.97, 0.0% | 0.680, 1.04(0.87-1.24) | 0.97, 0.0% | 0.71, 1.04(0.86-1.25) | 0.99, 0.0% |
| Population based | 3 | 0.971, 1.00(0.90-1.11) | 0.87, 0.0% | 0.686, 0.97(0.83-1.13) | 0.95, 0.0% | 0.53, 1.06(0.88-1.27) | 0.68, 0.0% |
| **Population** |  |  |  |  |  |  |  |
| North American | 3 | 0.918, 0.99(0.89-1.11) | 0.86, 0.0% | 0.889, 0.99(0.86-1.14) | 0.84, 0.0% | 0.79, 1.02(0.87-1.20) | 0.95, 0.0% |
| European | 4 | 0.550, 1.04(0.91-1.20) | 0.99, 0.0% | 0.881, 1.02(0.83-1.25) | 0.96, 0.0% | 0.40, 1.10(0.88-1.39) | 0.92, 0.0% |
